# Deflection Estimation of Truss Structures Using Inverse Finite Element Method

**DOI:** 10.3390/s23031716

**Published:** 2023-02-03

**Authors:** Zhaobo Zhang, Shuai Zheng, Hongnan Li, Liang Ren

**Affiliations:** State Key Lab of Offshore and Coastal Engineering, Dalian University of Technology, Dalian 116024, China

**Keywords:** deflection sensor, SHM, iFEM algorithm, distributed monitoring, truss structure

## Abstract

It is well recognized that strain and deflection data are important indexes to judge the safety of truss structures. Specifically, the shape sensing technology can estimate the deformation of a structure by exploiting the discrete strain data without considering the material property conditions. To fill the gap in which most of the methods in SHM (structural health monitoring) cannot be directly used to predict the displacement field, this paper proposed a novel inverse finite element method (iFEM) algorithm based on the equivalent stiffness theory. A deflection sensor is fabricated to focus on predicting the distributed deflection variation of the truss structure. The performance of the deflection sensor was evaluated by a calibration test and a stability test. Finally, it was applied to distributed deflection monitoring in the testing of truss structures. Results of all tests verify that the deflection sensor based on the i-FEM algorithm can predict the distributed deflection variation of the truss structure accurately, in real time, and dynamically.

## 1. Introduction

In recent years, large-span spatial structures consisting mainly of truss structures and mesh structures have developed rapidly due to their advantages of long span and lightweight [1]. During the construction stage, the structure is in a nonstationary state, and different construction sequences can lead to different results of stress redistribution. In addition, the construction steps, such as lifting, prearching, and unloading, will cause huge deformation of the structure; thus deflection is an important criterion for accepting the quality of the project at this stage. During the service stage, the structure is usually subjected to loss of stiffness due to the influence of adverse factors, such as vibration [2,3], corrosion [4,5,6], impact [7,8], and cracks [9,10], resulting in structural deformation. At this stage, deflection is an important basis for determining whether the structure is a safety risk. During the whole life cycle of a truss structure, it will be subjected to damage caused by human and environmental factors, thus posing safety hazards. Deflection is the direct physical quantity to evaluate the safety of the truss structure, and strain is the indirect physical quantity to evaluate the rod of the truss structure. At present, a nondestructive static method [11] and dynamic methods, such as the modal characteristics approach and the frequency domain approach [12], have become mature for estimating the strain of a space truss. Therefore, it is necessary to monitor the deflection of the truss structure in real time [13] using structural health monitoring consisting of a sensor subsystem, data acquisition subsystem, and data processing subsystem [14], which can realize multiple data synchronization, real-time acquisition, and processing. It also provides a key basis for the timely detection of structural damage and the localization of damage.

The mainstream methods for monitoring the deflection of truss structures are the electronic total station, laser distance measuring, static level method, RTK (real-time kinematic) measurement, and GPS (Global Positioning System) methods. Among them, the total station can process spatial data quickly and accurately through the infrared light and prism system. However, it cannot achieve real-time monitoring of structural deflection and distributed measurement of overall structural deflection because it requires manual operation. Similar to its measurement principle, the laser distance measuring method is to scan the spatial shape and structure of the object to obtain the spatial coordinates of the object through the triangulation principle. Similar to the electronic total station method, in the construction stage of the structure, the reference point is easy to be damaged, thus affecting the measurement accuracy. The static level method is a method of deflection measurement by a liquid cylinder, float, and precision level meter using the principle of connected liquid, but its working principle makes it impossible to monitor the dynamic deflection of the structure at multiple points. The RTK method based on the GNSS dynamic real-time differential measurement technology in the application of deflection monitoring has shown more and more superiority with its measurement continuity and real time, but it also needs a fixed reference station as a reference point so that it is difficult to apply at the construction stage of the structure. The GPS method is to obtain deflection data by processing the signals emitted by multiple satellites simultaneously by calculating different times of arrival at the measurement point through filtering algorithms simultaneously. It is widely used in deflection monitoring as a navigation and positioning system with the advantages of continuous and high accuracy. However, the roof or steel cover structure of a large-span spatial structure will obstruct the GPS signal transmission, and it is difficult to install and maintain during the operation stage of the structure. To sum up, for the deflection monitoring of the truss structures, most of the current methods in SHM cannot be used to predict the structural deflection field directly [15]. This is because these methods cannot simultaneously meet the needs of real-time monitoring, distributed monitoring, dynamic monitoring, and interchangeable sensors for monitoring truss structures. To overcome this problem, real-time shape sensing can play an important role in the development of SHM systems, especially for structures with complex loading conditions. The main benefit of shape sensing is that it allows for using the measured strain data to estimate the variation of the displacement field in the structure. Among various shape sensing methods, the typical strategies are the Ko displacement theory [16], modal method (MM) [17], and inverse finite element method (iFEM) [18]. Gherlone [15] compared the accuracy of these three methods through tests and concluded that the iFEM was more flexible and accurate than the other two techniques.

The iFEM is an advanced and powerful algorithm developed by Tessler and Spangler [19] for displacement and strain/stress monitoring of engineered structures. The iFEM discretizes the whole structure into suitable inverse finite elements (beam, plate, shell, and solid elements), and the only input data are the strain of each element. It utilizes the least squares methodology to minimize differences between measured and numerical strains. This process leads to a system of equations in a matrix form. By solving the matrix system, the deflections at each node of iFEM elements can be determined in real time. Since the iFEM algorithm uses the Tikhonov regularization, which is manifested by constraint (regularity) terms that ensure a certain degree of solution smoothness, the accuracy of the solution can be guaranteed [20]. The essence of this inverse problem of the iFEM is reflected by Tessler and Spangler, who defined it as the generation of the displacement field of a structure by means of strain data collected by sensors placed at different locations of the structure.

The existing shape sensing methods [21] all require the shape sensing auxiliary structure to be installed on the structure to be measured so that the shape sensing auxiliary structure and the structure to be measured are deformed simultaneously, and the measurement of the auxiliary structure is measured to achieve the deflection monitoring of the structure to be measured. However, in actual engineering, there may not be space to install auxiliary structures, and auxiliary structures may change the original stress distribution of the structure. Therefore, the development of a deflection sensor for a distributed, real-time, and dynamic measurement of truss structures without the aid of auxiliary structures is necessary for SHM in large-span spatial structures.

In this paper, a novel deflection sensor is proposed to predict the deflection of the truss structure. The sensor reduces the truss structure to a beam element by the equivalent stiffness theory and divides it into several two-node inverse beam elements by the inverse finite element theory. The acquired strain information on each inverse element is converted into deflection information by the built-in algorithm of the deflection sensor. The accuracy of the deflection sensor and its stability under the influence of residual stresses are confirmed by a calibration test and a cyclic test. Finally, according to the various loading types in practical engineering, a deflection test based on steel trusses is designed to evaluate the accuracy and stability of the proposed deflection sensor for a distributed, real-time, and dynamic measurement of truss structures.

## 2. Inverse Finite Element Formulation of the Truss Structure

### 2.1. The Principle of Equivalent Stiffness

The force characteristics of the truss structure are that when the loading acts on the truss nodes, each element of the structure is only subjected to axial force, and there is no bending moment and shear force. However, the actual truss member is subject to bending moment and shear force because the nodes are welded, but nonideal hinge nodes, which meet the basic conditions of an iFEM algorithm.

As an important constituent member of the truss structure, the chord rod has numerous nodes connected to it, and the nodes are surrounded by complex force areas, so it is difficult to measure the strain accurately. Moreover, its boundary conditions are extremely complicated since it is subjected to complex forces. Therefore, it is impossible to monitor the deformation of the truss structure by calculating the deformation of one chord bar of the truss structure with the iFEM algorithm directly.

The upper chord of the truss structure is a pure compression bar, and the lower chord is a purely tensioned bar, which meets the calculation conditions of the iFEM algorithm. Thus, the truss is divided into multiple inverse elements according to the span for shape sensing calculation. The displacement field distribution of each inverse element can be obtained in real time to realize real-time, distributed, and dynamic deflection monitoring of the truss structure.

**Figure 1:**
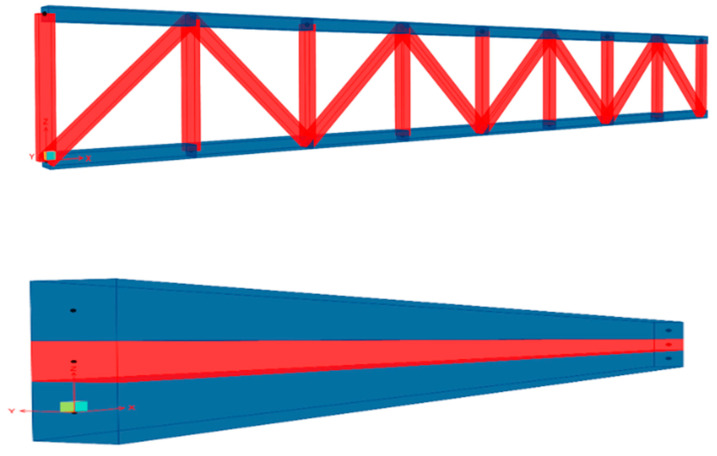
Simplification of truss structure to beam.

Since the truss is evolved from the beam, according to the definition of structural mechanics, the underutilized material near the neutral axis of the beam is hollowed out to obtain the truss. According to this theory, in order to calculate the deflection of the truss structure accurately, it is necessary to simplify the truss structure into a beam structure. As shown in Figure 1, the truss structure is simplified as a beam, the upper chords of the truss are simplified as the upper surface of the beam, the lower chords are simplified as the lower surface of the beam, and the web rods are simplified as the side surface of the beam.

The equation for the midspan deflection of a beam of homogeneous elastic material in the mechanics of materials is:

(1) $$f=S\frac{Ml_{0}^{2}}{EI}$$

(2) $$E=\frac{\sigma }{\varepsilon }=\frac{F}{A\varepsilon }$$

where *S* and *M* are the coefficient related to the load type and boundary conditions, and *l_0_* is the length of the beam. *EI* is the flexural stiffness of the cross section. Among them, *E* is the ratio coefficient of stress(*σ*) and strain(*ε*) during the elastic deformation phase of the material. It is only related to the chemical composition of the material; *I* is the moment of inertia of the section measuring the bending resistance of the section. In addition, *σ* is the force *F* exerted on unit area *A*. The inverse finite element algorithm is only related to strain and not to material properties and load. For the homogeneous elastic materials, it is known as Equation (1) in which the deflection *f* is only related to the flexural stiffness *EI* when the external load and length are determined. It is known as Equation (2) in which the modulus of elasticity *E* is only related to the strain, external load, and cross section. Further, the deflection *f* is only related to the moment of inertia *I* of the cross section, which is a geometric parameter measuring the flexural capacity of the cross section and is the integral of the quadratic product of the distance associated with the area of the cross section; then, it is only related to the properties of the cross section. According to the theory, the truss structure can be simplified to a beam element. The height of the beam after simplification is defined as

(3) $$H=\frac{h_{1}+h_{2}}{2}+h_{3}$$

where *h*_1_ is the equivalent height of the upper chord of the truss structure, *h*_2_ is the equivalent height of the lower chord, and *h*_3_ is the equivalent height of the web. It can be described as follows:

(4) $$h_{3}=\frac{A\times \cos^{2}\theta \times h_{a}\times b_{a}\times L_{a}+B\times h_{b}\times b_{b}\times L_{b}}{h_{c}\times b_{c}\times L_{c}}\times h_{c}$$

where *A* and *B* are the number of diagonal webs and vertical webs, respectively; *h_a_*, *h_b_*, and *h_c_* are the height, width, and length of the diagonal web section, respectively; and *L_a_*, *L_b_*, and *L_c_* are the height, width, and length of the section of the whole truss, respectively.

Then the truss structure is simplified to beam by the equivalent stiffness theory. In order to avoid the interference of the complex stress area near the nodes to the iFEM algorithm, the length of each span is a two-node inverse-beam element according to the span of the truss.

### 2.2. The Principle of iFEM Algorithm

**Figure 2:**
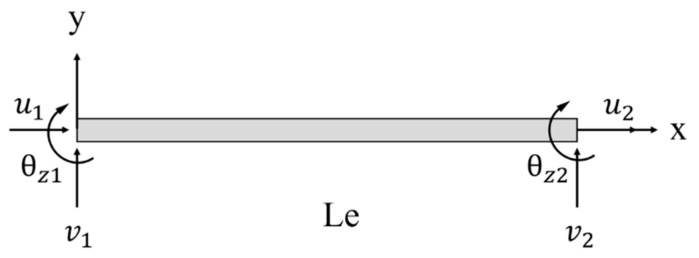
Two-node inverse-beam element.

As shown in Figure 2, the two-node inverse-beam element is developed based on the classical beam theory utilizing the iFEM methodology as its mathematical framework for the truss structure. The element contains two nodes; each node has a three-displacement degree of freedom and an element thickness of 2*h*.

Based on the classical beam theory, the displacement fields of the two-node inverse-beam element are defined as

(5) $$u_{x}(x,y,z)=u(x)-y\theta_{z}(x)$$

(6) $$u_{y}(x,y,z)=v(x)$$

where *u_x_* is the displacement of each point along the x direction, *u_y_* is the displacement of each point along the y direction, *u* and *v* are the translational degrees of freedom along the *x* and *y* directions, and *θ_z_* is the rotational degrees of freedom along the z direction.

The element shape function and the element end node degrees of freedom are used to express the element internal displacement function as follows:

(7) $$u(x)=\sum_{i=1,2}N_{i}u_{i}$$

(8) $$v(x)=\sum_{i=1,2}Q_{i}v_{i}+\sum_{i=1,2}M_{i}\theta_{zi}$$

(9) $$\theta_{z}(x)=\frac{\partial v(x)}{\partial x}=\sum_{i=1,2}Q_{i,x}v_{i}+\sum_{i=1,2}M_{i,x}\theta_{zi}$$

where

(10) $$N_{1}=1-\xi$$

(11) $$N_{2}=\xi$$

(12) $$Q_{1}=2\xi^{3}-3\xi^{2}+1$$

(13) $$Q_{2}=3\xi^{2}-2\xi^{3}$$

(14) $$M_{1}=L(\xi^{3}-2\xi^{2}+\xi )$$

(15) $$M_{2}=L(\xi^{3}-\xi^{2})$$

where *N_i_*, *Q_i_*, and *M_i_* are the element shape function, *ξ* = *x*/*L* ∈ [0, 1], which is a dimensionless local coordinate. In Equations (8) and (9), the coupled interpolation of the deflection and section rotation allows the element to achieve a cubic polynomial interpolation of the deflection field using only two nodes, which ensures the accuracy of the displacement field.

Combining Equation (5) with Equation (15) yields

(16) $$\varepsilon_{x}=\frac{\partial u_{x}}{\partial x}=\varepsilon (u_{e})-yk(u_{e})=B_{b}u_{e}-yB_{m}u_{e}$$

where

(17) $$u_{e}={\left[u_{1}\;v_{1}\;\theta_{z}{}_{1}\;u_{2}\;v_{2}\;\theta_{z}{}_{2}\right]}^{T}$$

(18) $$B_{b}=\left[N_{1,x}\;0\;0\;N_{2,x}\;0\;0\right]$$

(19) $$B_{m}=\left[0\;Q_{1,xx}\;M_{1,xx}\;0\;Q_{2,xx}\;M_{2,xx}\right]$$

where **ε**(**u***_e_*) and **k**(**u***_e_*) are the axial strain and bending curvature due to tension.

**Figure 3:**
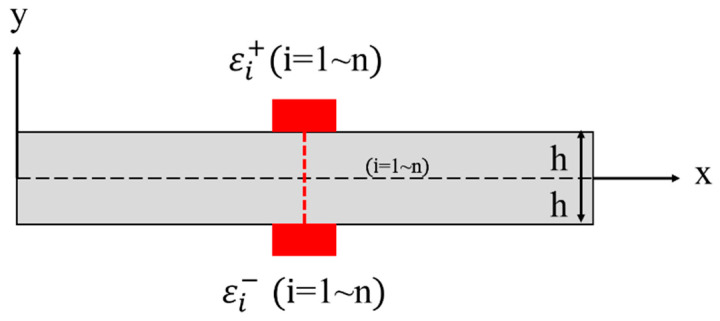
Discrete surface strain measured by strain sensors within two-node inverse-beam element.

Strain measurements at discrete locations after structural deformation are essential for the inverse finite element solution. The placement scheme of the sensor is shown in Figure 3. Two strain sensors are placed on the top and bottom surfaces of each discrete element. The measured values **ε*_i_*** and **k***_i_* corresponding to the axial strain **ε**(**u***_e_*) and the bending curvature **k**(**u***_e_*) can be expressed as

(20) $$\varepsilon_{i}=\frac{\varepsilon_{i}^{+}+\varepsilon_{i}^{-}}{2}$$

(21) $$k_{i}=\frac{\varepsilon_{i}^{+}-\varepsilon_{i}^{-}}{2h}$$

where, *i* (*i* = 1, … , *n*) is the location of the strain measurement point inside the element.

The inverse finite element is based on the least squares variational principle to solve the inverse strain–displacement problem of the structure. The method minimizes the mean square error sum of the theoretical and measured strains by solving the generalized extremum to find the optimal nodal degree of freedom vector. For the two-node inverse-beam element, considering tensile and bending deformations, the least-squares generalized function $\Phi_{e}{(u}_{e})$ is defined as

(22) $$\Phi_{e}(u_{e})=\omega_{b}{\left\|\varepsilon (u_{e})-\varepsilon \right\|}^{2}+\omega_{m}{\left\|k(u_{e})-k\right\|}^{2}$$

The two-parametric number in Equation (22) can be expressed as a normalized Euclidean parametric number as follows:

(23) $$\omega_{b}{\left\|\varepsilon (u_{e})-\varepsilon \right\|}^{2}=\frac{1}{L_{e}}\sum_{i=1}^{n}\int_{\frac{(i-1)L_{e}}{n}}^{\frac{iL_{e}}{n}}\omega_{bi}{\left({\left(\varepsilon (u_{e})\right)}_{i}-\varepsilon_{i}\right)}^{2}dx$$

(24) $$\omega_{m}{\left\|k(u_{e})-k\right\|}^{2}=\frac{{\left(2h\right)}^{2}}{L_{e}}\sum_{i=1}^{n}\int_{\frac{(i-1)L_{e}}{n}}^{\frac{iL_{e}}{n}}\omega_{mi}{\left({\left(k(u_{e})\right)}_{i}-k_{i}\right)}^{2}dx$$

where *L_e_* is the element length, (2*h*) is the section height, and n is the number of measurement points inside the element. The scale factor is the (2*h*)^2^/*L_e_* in Equation (24). The two-parametric numbers are expressed in dimensionless form uniformly, and their values are determined by Equations (20)–(22).

If the measurement data are missing at the M-point, the corresponding weighting factor takes a smaller value (λ = 10^−8^), and the two-parameter number in Equation (22) takes its reduced form as follows:

(25) $$\begin{array}{ll} \omega_{b}{\left\|\varepsilon (u_{e})-\varepsilon \right\|}^{2}= & \frac{1}{L_{e}}(\sum_{i=1}^{m-1}\int_{\frac{(i-1)L_{e}}{n}}^{\frac{iL_{e}}{n}}\omega_{bi}{\left({\left(\varepsilon (u_{e})\right)}_{i}-\varepsilon_{i}\right)}^{2}dx+\int_{\frac{(m-1)L_{e}}{n}}^{\frac{mL_{e}}{n}}\lambda {\left(\varepsilon (u_{e})\right)}^{2}\\ & +\sum_{i=m+1}^{n}\int_{\frac{(i-1)L_{e}}{n}}^{\frac{iL_{e}}{n}}\omega_{bi}{\left({\left(\varepsilon (u_{e})\right)}_{i}-\varepsilon_{i}\right)}^{2}dx) \end{array}$$

(26) $$\begin{array}{ll} \omega_{m}{\left\|k(u_{e})-k\right\|}^{2}= & \frac{{\left(2h\right)}^{2}}{L_{e}}(\sum_{i=1}^{m-1}\int_{\frac{(i-1)L_{e}}{n}}^{\frac{iL_{e}}{n}}\omega_{mi}{\left({\left(k(u_{e})\right)}_{i}-k_{i}\right)}^{2}dx+\int_{\frac{(m-1)L_{e}}{n}}^{\frac{mL_{e}}{n}}\lambda {\left(k(u_{e})\right)}^{2}\\ & +\sum_{i=m+1}^{n}\int_{\frac{(i-1)L_{e}}{n}}^{\frac{iL_{e}}{n}}\omega_{mi}{\left({\left(k(u_{e})\right)}_{i}-k_{i}\right)}^{2}dx) \end{array}$$

Substituting Equations (25) and (26) into Equation (22), Equation (22) can be minimized concerning the nodal displacement DOF by finding the extremum, giving rise to

(27) $$\frac{\partial \Phi_{e}(u_{e})}{\partial u_{e}}=k_{e}u_{e}-f_{e}=0$$

where **k***_e_* is the elementary coefficient matrix, **f***_e_* is the elementary strain vector, and **u***_e_* is the elementary nodal degree of freedom vector. The **k***_e_* can be explicitly written as follows:

(28) $$k_{e}=\sum_{i=1}^{n}\int_{\frac{(i-1)L_{e}}{n}}^{\frac{iL_{e}}{n}}(\omega_{bi}{\left(B_{b}\right)}^{T}B_{b}+\omega_{mi}{\left(2h\right)}^{2}{\left(B_{m}\right)}^{T}B_{m})dx$$

The **f***_e_*, however, is dependent on the measured strain values. It is defined as follows:

(29) $$f_{e}=\sum_{i=1}^{n}\int_{\frac{(i-1)L_{e}}{n}}^{\frac{iL_{e}}{n}}(\omega_{bi}{\left(B_{b}\right)}^{T}\varepsilon_{i}+\omega_{mi}{\left(2h\right)}^{2}{\left(B_{m}\right)}^{T}k_{i})dx$$

From Equations (28) and (29), it can be seen that the cell coefficient matrix **k***_e_* is a constant matrix and is related only to the geometry of the elements (*L_e_* and *h*) and the weighting factors (*b* and *m*). The element strain vector **f***_e_* is related to the measured strain data.

After establishing the matrix equations of the elements in the local coordinate system, the discrete element matrix equations are integrated into the total system equations using the coordinate transformation matrix.

(30) $$K=\sum_{e=1}^{nel}{(T_{e})}^{T}k_{e}T_{e}$$

(31) $$F=\sum_{e=1}^{nel}{\left(T_{e}\right)}^{T}f_{e}$$

(32) $$U=\sum_{e=1}^{nel}(T_{e}{)}^{T}u_{e}$$

(33) KU=F

where

(34) $$T_{e}=\left[\begin{array}{cccccc} \cos\theta & \sin\theta & 0 & 0 & 0 & 0\\ -\sin\theta & \cos\theta & 0 & 0 & 0 & 0\\ 0 & 0 & 1 & 0 & 0 & 0\\ 0 & 0 & 0 & \cos\theta & \sin\theta & 0\\ 0 & 0 & 0 & -\sin\theta & \cos\theta & 0\\ 0 & 0 & 0 & 0 & 0 & 1 \end{array}\right]$$

where **K** is the coefficient matrix of the overall coefficients, **F** is the vector of the overall strain, **U** is the vector of the degrees of freedom of the overall nodes, **T***_e_* is the coordinate transformation matrix, *e* is the number of the inverse element, and *θ* is the angle between the local coordinate system x-axis and the overall coordinate system X-axis, with the clockwise rotation of the x-axis to the X-axis as the positive direction.

The coefficient matrix **K** in Equation (33) is a singular matrix, which cannot achieve the solution of the vector of the degrees of freedom of the nodal. To eliminate matrix singularity, considering the problem-specific displacement boundary conditions, Equation (33) can be rewritten by its reduced form as

(35) $$K_{R}U_{R}=F_{R}$$

where **K**_R_ is always nonsingular and, accordingly, reversible. Once the discretization strategies are determined, the matrix **K**_R_ remains unchanged, and its inverse needs to be calculated only once during the entire monitoring process. The matrix F_R_ is associated with the in situ strain data and needs to be updated in real time. In the solution of the unknown DOF vector **U**_R_, only a simple linear operation is required, directly determining high computation efficiency, and can meet dynamic monitoring for the iFEM [20]. In addition, by substituting the node degrees of freedom (**u***_e_*) of each two-node inverse-beam element into Equations (7)–(9), the displacement field of the truss structure can be monitored.

## 3. Performance Study of the Deflection Sensor

Since the iFEM algorithm based on the inverse finite element principle and the equivalent stiffness principle can convert the strain information into the deflection information of the truss structure, it can be used as a deflection sensor for monitoring the deflection of the truss structure. The workflow of the deflection sensor is as follows: first, the FBG stress sensors measure the strain of the truss; second, the strain values are converted into displacement values (deflection values) by the iFEM algorithm. In this section, to evaluate its performance, the deflection sensor based on the iFEM algorithm is used for the calibration test and stability test.

### 3.1. Calibration Test

The deflection sensor based on the iFEM algorithm is used for the following calibration test to evaluate the predictive performance of the sensor for the deflection monitoring of the truss structure. Tests are conducted on a truss of 6000 mm in length and 440 mm in height with Young’s modulus E of 210 GPa. The truss is divided into eight two-node inverse-beam elements based on a length of 750 mm per span; FBG sensors were fabricated by standard monomode optical fiber with a diameter of 4 mm, a length of 90 mm, a standard distance of 60 mm, a range of ±1500 με, a central wavelength range of 1510–1590 nm, a sensitivity of 4.8 pm/με, a resolution of 1με, and an accuracy of 0.1% F.S. A uniform periodic grating is used to determine the average strain at the scale length, and the FBG (fiber Bragg grating) strain sensors are installed on the top and bottom surfaces at the center of each inverse element to measure the strain. A micrometer is placed at the midspan of the truss to measure the actual deflection as the standard value. In the calibration test, the truss is loaded continuously from 0 to 60 kg at an interval of 10 kg.

**Figure 4:**
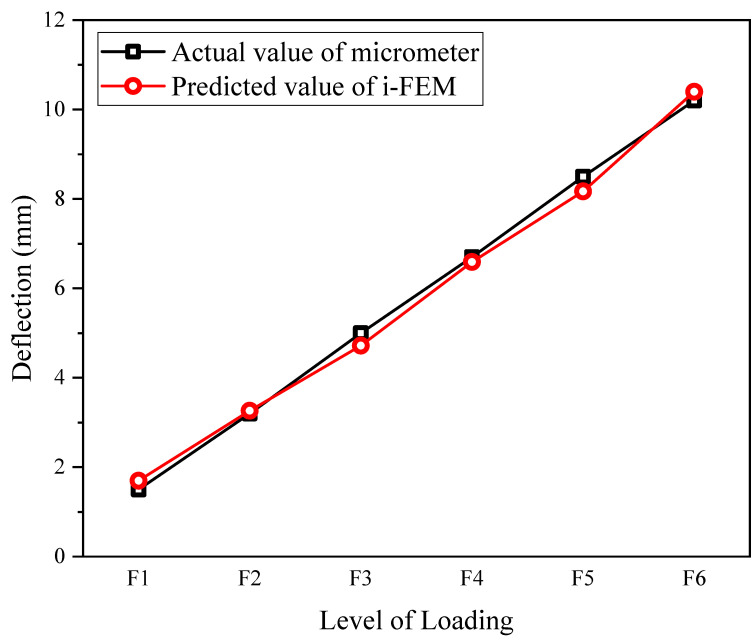
Results of calibration.

As shown in Figure 4, the deflection distribution under each level of loading appears to a horizontal line and varies linearly, which agrees well with the actual loading situation and the linear elastic characteristics of the material. The deviations of the predicted deflection by the iFEM algorithm from the measured deflection by the micrometer under six levels of loading are 0.19, 0.06, 0.28, 0.11, 0.33, and 0.19 mm, respectively. The results demonstrate that the deflection sensor based on the iFEM algorithm for a truss structure is highly accurate in predicting the deflection.

### 3.2. Stability Test

**Figure 5:**
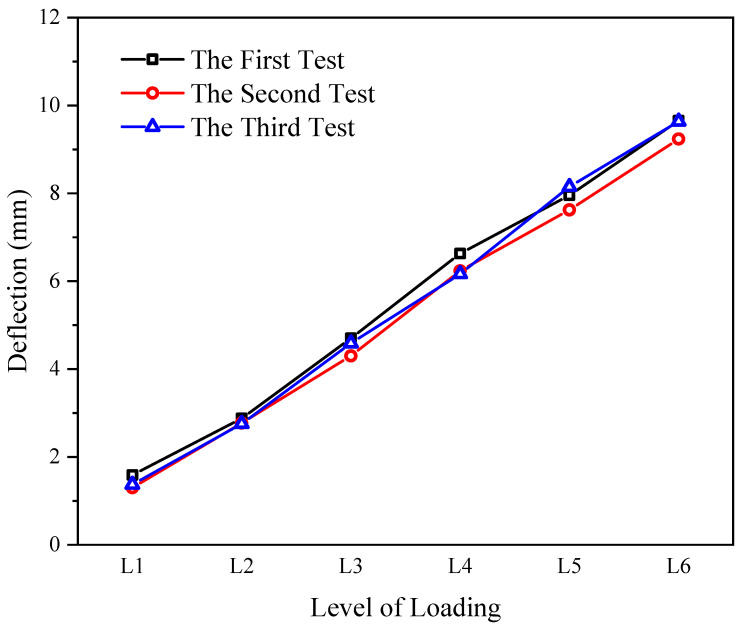
Deflection values under cyclic loading.

The residual stresses inevitably exist in steel structures under cyclic loading. Since the essence of the iFEM algorithm is to transfer the strain to the displacement, complex residual stress conditions may affect the accuracy of dynamic monitoring. To investigate the accuracy and stability of the deflection sensor under the effect of residual stresses to meet the requirements of dynamic monitoring for truss structure, the stability test was conducted on the truss structure with a six-level loading and a constant loading of 30 s after each level with three cycles. The strain data recorded by the transducer under each loading level were recorded separately, after which the strain data were converted into predicted deflection values by the two-node inverse-beam element based on the iFEM algorithm. The predicted deflection values under three tests are compared to verify the stability of the deflection sensor under the influence of residual stress. The data from the three stability tests are shown in Figure 5.

The average deviation under six levels of loading for the three tests is 0.28, 0.12, 0.39, 0.46, 0.52, and 1.42 mm, respectively. The maximum deviation value is only 0.52 mm. The stability test confirmed that the deflection sensor remains highly accurate and stable under the influence of residual stresses and verified that the deflection sensor meets the requirements of truss monitoring for dynamic monitoring.

## 4. Application of the Deflection Sensor in the Truss Structure

In practical engineering, the truss structures may be subjected to multitype loading, including the central loading during the prearch period and the eccentric loading and combined loading due to the environmental factors at the service stage. In this section, to study the accuracy of the deflection sensor based on the iFEM algorithm in predicting the deflection of a truss structure in practical engineering, a multicase of loading tests, such as central loading, eccentric loading, and combined loading, is conducted on the truss structure.

### 4.1. Testing Setup

**Figure 6:**
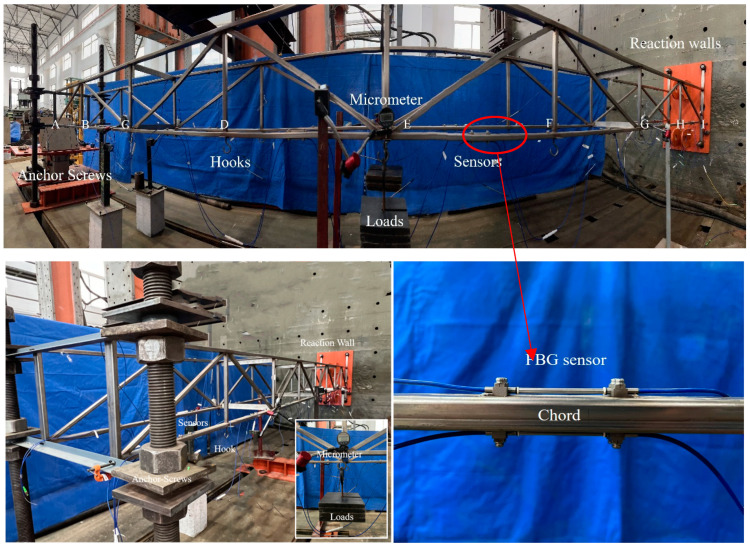
The test setup.

The size parameters and structural forms of the double-bay clamped–clamped truss is expressed in Figure 6, where Young’s modulus is 210 GPa, Poisson’s ratio is 0.3, the length of the clamped–clamped truss is 6000 mm, and the height of the clamped–clamped truss is 440 mm. The length of each span is 750 mm, the upper and lower chords have a rectangular cross section of 20 mm height and 40 mm width, and the vertical webs and diagonal webs have a rectangular cross section of 20 mm height and 20 mm width. The ends of the double-bay truss are clamped by reaction walls and anchor screws. Two micrometers are placed at the midspan of each truss to capture the measured deflection data, and the loading is applied by hooks that are welded at the loading points of the lower chords. The FBG strain sensors and the data acquisition equipment are used to measure and analyze strain data.

**Figure 7:**
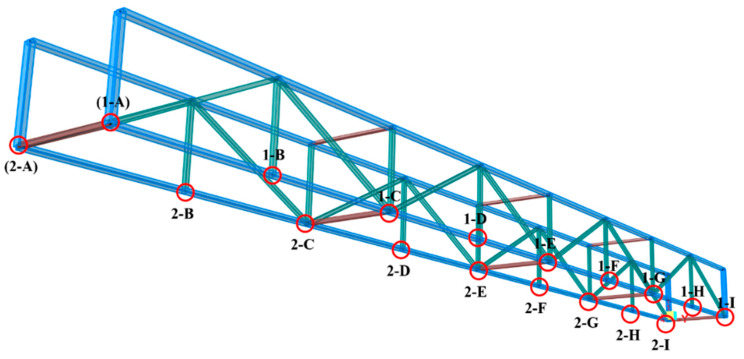
The distribution of the loading points.

The distribution of loading points is shown in Figure 7. The details of four loading cases are shown in Table 1.

As shown in the table above, a concentrated force is applied at the loading points E and F of the truss structure for cases 1 and 2, respectively. A set of concentrated forces is applied at the loading points E and F of the truss structure for case 3. A set of concentrated forces is applied to the loading points D, E, and F of the truss structure for case 4. The level of loading increased continuously from 0 kg with an interval of 10 kg.

### 4.2. Testing Results

**Figure 8:**
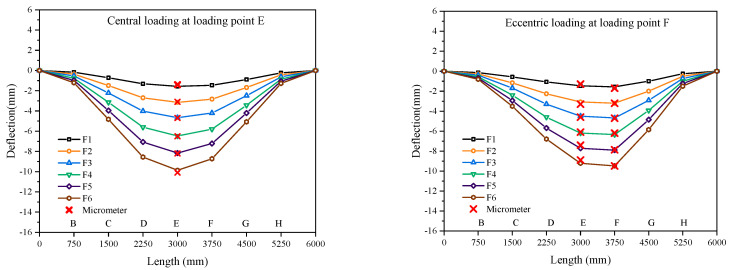

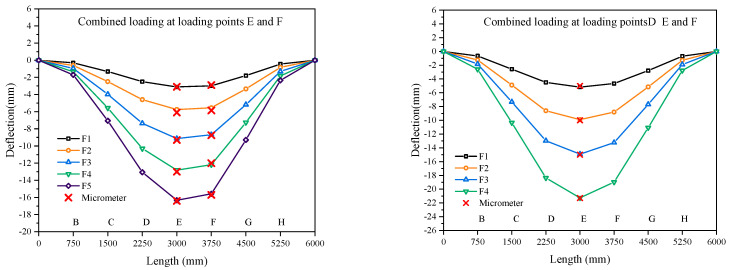
Schematic diagram of the shape of the truss.

A comparison of deflection results for the four cases are shown in Figure 8, with the horizontal coordinate representing the distribution of the loading points and the vertical coordinate representing the variation value of deflection. Among them, the colored curves represent the shapes of the truss structure predicted by the deflection sensor based on the iFEM algorithm under all levels of loading, and the red crosses represent the measured deflection values of the micrometer placed in the midspan of the truss structure.

As shown in Figure 8, the results clearly show the displacement of the nodes of each inverse element as the applied loading increases. These displacements are extracted and plotted as changes in the shape of the truss structure. These demonstrate that the deflection sensor based on the iFEM algorithm is suitable for the distributed deflection measurement of truss structures in real time.

In addition, in the four cases, the results of the deflection predicted by the deflection sensor based on the iFEM algorithm are used to create a comparison chart with the measured deflection from the micrometer. It can be clearly seen that the predicted results are in high agreement with the measured results. The deflection measurements obtained by the micrometer and the inverse finite element method are shown in Table 2. The maximum deviation between the measured deflection values of the micrometer and the predicted deflection values of the deflection sensor under all levels of loading are 0.3, 0.3, 0.6, and 0.7 mm, respectively.

Finally, according to the theory of material mechanics, the maximum value of deflection of a truss structure is only related to the moment of inertia of the span section, the modulus of elasticity of the material, and the type of loading on the truss. Combined with the general specification for steel structures, it can be obtained that for the parallel chord truss, the maximum value of deflection is about 50 mm. The average deviation and deviation rate of four cases in the test are shown in Table 2. Among them, the average deviations for the four cases are only 0.19, 0.11, 0.23, and 0.6 mm, which are only 0.39%, 0.22%, 0.48%, and 1.23% of the maximum deflection of 50 mm, respectively. These demonstrate that the deflection sensor based on the iFEM algorithm has the stability and accuracy for the deflection measurement of truss structures.

In summary, the sources of deviations in the tests are noise interference from the sensors and unavoidable fabrication errors in the steel structure, such as stress concentrations at the welds and welding errors in the structure. The testing results show that the deflection sensor based on the i-FEM algorithm can predict the distributed deflection variation of the truss structure accurately, in real time, and dynamically. On the other hand, this sensor can accurately obtain the strain field and displacement field information of the structure by simply acquiring the structural strain data through the sensor, which fills the gap of the SHM system’s inability to predict the structural displacement field to a certain extent.

## 5. Conclusions

In view of the deficiency in which most existing deflection monitoring methods cannot meet the requirements of real-time, dynamic, and distributed monitoring for truss structures of large-span spatial structures and the composition of truss structures is complex, the existing iFEM algorithm based on a beam element cannot be directly applied to the truss structures without an auxiliary monitoring method. In this paper, a novel iFEM algorithm is proposed to predict the deflection of the truss structure. The algorithm reduces the truss structure to a beam element by the equivalent stiffness theory and divides it into several two-node inverse-beam elements by the inverse finite element theory. Its strain-deflection transfer mechanism is analyzed in detail and used as a deflection sensor for truss structures. A calibration test and a stability test are carried out on a truss structure to assess the measurement performance of the deflection sensor. Finally, the deflection sensor is applied to the distributed deflection monitoring of a truss structure. The results demonstrate that the deflection sensor based on the iFEM algorithm can predict the distributed deflection variation of the truss structure accurately, in real time, and dynamically. In addition, the FBG sensors used in the deflection sensor have higher cost performance compared with the total station. Moreover, the FBG sensors are an optical sensor, which can keep operating in bad weather.

There are several strengths in our study. Since the strain sensors are built in, the deflection sensor is usually deployed in the midspan of the rods. Meanwhile, this position is an important location to obtain the structural stress field. This deflection sensor can also obtain the stress information of some bars. This study has several limitations. First, trusses of various types and sections will be applied in practical engineering, so the simplification principle proposed for the trusses in this paper will have certain limitations. Second, the spatial grid system is often used in long-span spatial structures. The interconnected trusses will provide additional constraints and boundary conditions, which may complicate the prediction of deflection under dynamic loads, such as wind and vibration. In future work, there are two parts to consider: the first part is to study the accuracy of the deflection prediction of a sensor under dynamic load, such as wind vibration; the second part is to study the equivalent simplification principle of a truss with different sections and expand the application range of a deflection sensor in large-span spatial structures.

## Figures and Tables

**Table 1:** The loading position and the level of loading in each case.

Case Number	Loading Position	Level of Loading	Type of Loading
1	E	F1-F6	Central loading
2	F	F1-F6	Eccentric loading
3	E-F	F1-F5	Combined loading
4	D-E-F	F1-F4	Combined loading

**Table 2:** Detailed list of deflections.

Central loading		E Point					
		Micrometer	iFEM	Deviation			
	F1	−1.5	−1.57	0.07			
	F2	−3.2	−3.26	0.06			
	F3	−5.0	−4.67	0.30			
	F4	−6.7	−6.47	0.23			
	F5	−8.2	−7.97	0.23			
	F6	−10.2	−10.40	0.20			
Eccentric loading		E Point			F Point		
		Micrometer	iFEM	Deviation	Micrometer	iFEM	Deviation
	F1	−1.3	−1.28	0.01	−1.7	−1.57	0.13
	F2	−3.3	−3.21	0.09	−3.2	−3.21	0.00
	F3	−4.6	−4.91	0.31	−4.7	−4.68	0.03
	F4	−6.1	−6.10	0.00	−6.2	−6.35	0.15
	F5	−7.4	−7.47	0.06	−7.9	−7.90	0.03
	F6	−8.9	−9.06	0.16	−9.5	−9.48	0.01
Combined loading– E and F		E Point			F Point		
		Micrometer	iFEM	Deviation	Micrometer	iFEM	Deviation
	F1	−3.1	−2.98	0.13	−2.9	−2.98	0.08
	F2	−6.1	−6.27	0.17	−5.8	−5.54	0.31
	F3	−9.5	−9.41	0.10	−8.8	−8.68	0.07
	F4	−13.0	−12.42	0.58	−12.0	−12.18	0.18
	F5	−16.4	−16.20	0.20	−15.7	−15.58	0.12
Combined loading– D, E, and F		E Point					
		Micrometer	iFEM	Deviation			
	F1	−5.1	−4.61	0.49			
	F2	−10.0	−9.38	0.62			
	F3	−14.9	−14.35	0.55			
	F4	−21.3	−20.60	0.70			
